# Molecular enhancement of sentinel node evaluation

**DOI:** 10.1186/1479-5876-13-S1-K4

**Published:** 2015-01-15

**Authors:** Alistair J Cochran

**Affiliations:** 1 Departments of Pathology, Laboratory Medicine and Surgery, David Geffen School of Medicine at UCLA, Los Angeles, California, USA

## 

Lymphatic mapping and sentinel node biopsy are widely used to stage and manage patients with intermediate and thicker primary cutaneous melanoma. *Likelihood* of sentinel node metastases can be estimated from patients’ demography and primary tumor characteristics, but is precisely *determined* only by microscopic evaluation of excised node(s). Molecular/Genetic techniques, such as RT-PCR, Gene Expression Microarray, detection of metastasis-associated gene signatures and gene sequencing are likely to increase precision in future. Recent 10 year results of Multicenter Selective Lymphadenectomy Trial 1 (MSLT1) [1] confirm that biopsy-based management prolongs disease free survival for all node-positive patients (P=0.01-0.03) and significantly increases 10 year distant disease-free survival (P=0.006) and melanoma-specific survival (P=0.02) for node-positive patients with intermediate thickness primaries. The trial also confirmed very clearly that the presence or absence of sentinel node metastases is best determined by close pathological evaluation of sections from nodal tissues, stained conventionally and by immunohistochemistry (S-100 protein, Mart-1, HMB-45). Patients with sentinel node metastases have a significantly unfavorable outcome that correlates with the amount and disposition of nodal tumor as assessed by microscopy, immunohistochemistry and morphometry [2]. Here again precision will be increased by the parallel application of molecular and genetic tests. The accuracy of appropriate patient assignment to early surgical management will be dramatically increased by the addition of molecular testing, more precisely identifying the patients most likely to respond to standard and evolving therapies
